# Supplementary material for: Catalyst switch strategy enabled a single polymer with five different crystalline phases
Source: Nat Commun. 2023 Nov 20;14:7559. doi: 10.1038/s41467-023-42955-3 (PMC10662249; doi:10.1038/s41467-023-42955-3)
Supplement: Supplementary file 1 — Supplementary Information [file 41467_2023_42955_MOESM1_ESM.pdf]

Supplementary Information

Catalyst Switch Strategy Enabled A Single Polymer with Five Different Crystalline Phases

Pengfei Zhang[1], Viko Ladelta[1], Edy Abou-hamad[2], Alejandro J. Müller[3] and Nikos Hadjichristidis[1]*

[1]Polymer Synthesis Laboratory, KAUST Catalysis Center, Chemistry Program, Physical Sciences and Engineering Division, King Abdullah University of Science and Technology (KAUST), Thuwal 23955, Saudi Arabia.

[2]Imaging and Characterization Core Lab, King Abdullah University of Science and Technology (KAUST), Thuwal 23955, Saudi Arabia.

[3]Department of Polymers and Advanced Materials, Physics, Chemistry and Technology, Faculty of Chemistry, University of the Basque Country UPV/EHU, Paseo Manuel de Lardizabal 3, 20018, Donostia-San Sebastián, Spain

Email: nikolaos.hadjichristidis@kaust.edu.sa

Table of Content

Materials S3

Instruments S3

Figures, Tables, and Discussions S5

Supplementary Figures 1-7 S5

Supplementary Table 1 S9

Supplementary Figures 8-16 S10

Supplementary Discussion: Solid-state NMR analysis S15

Supplementary Discussion: FTIR analysis S18

Supplementary Figures 26-36 S20

References S27

Materials

All solvents were purchased from VWR (HPLC grade) and used as received unless otherwise stated. Deuterated toluene (toluene-d_8, 99.8% D) and $CDCl_3$ were purchased from Sigma-Aldrich and were used for NMR. 1,2,4-trichlorobenzene (TCB; > 99%, anhydrous), 1,4-dioxane (anhydrous, > 99.9%), 1-tert-butyl-4,4,4-tris(dimethylamino)-2,2-bis[tris(dimethylamino)-phosphoranylidenamino]-2λ^5,4λ^5-catenadi(phosphazene) (^{t}BuP$_4$, 0.8 M solution in hexane), acetic acid (99.5%), calcium hydride (CaH_2, 95%), trimethylsulfoxonium iodide (> 98%), benzyltributylammonium chloride (98%), triethylborane (TEB) in hexane (1.0 M), trimethylamine-N-oxide dihydrate (TAO.2H_2O; > 99%), sodium hydride (NaH), and trifluoroacetic acid (TFAA) were purchased from Sigma-Aldrich and used as received. Toluene and tetrahydrofuran (THF) were dried by distillation over CaH_2 (overnight) and ^{n}butyllithium (3 hours). *ε*-caprolactone (CL, 99%) from Sigma-Aldrich was distilled twice over CaH_2 under a dynamic vacuum. L-lactide (LLA) (99%, Alfa-Aesar) and glycolide (GL, 99% Sigma-Aldrich) were recrystallized from EtOAc three times, dissolved in anhydrous 1,4-dioxane, cryo-evaporating the 1,4-dioxane, followed by drying under vacuum overnight. Diphenyl phosphate (DPP; 99%, Sigma-Aldrich) was also dried by cryo-evaporation in anhydrous 1,4-dioxane. Ethylene oxide (EO, anhydrous, >99.5 %, Sigma-Aldrich) was first transferred from a steel cylinder to a Schlenk flask equipped with a liquid nitrogen cryo bath and dried by stirring with NaH in an ice–water bath for 4 h. The EO was distilled into a pre-calibrated Schlenk tube containing ^{n}butyllithium (1.6 M in hexanes, Sigma-Aldrich). The tube was placed in an ice–water bath, and EO was stirred for another 1 h. Sn(Oct)$_2$, (95%, Sigma-Aldrich) was distilled twice over anhydrous $MgSO_4$ and activated 4 Å molecular sieves followed by azeotropic distillation with dry toluene. All monomers, solvents, and catalysts for polymerizations were stored under argon (Ar) in a glove box (LABmasterproSP, MBraun, Germany).

Instruments

Liquid-state Nuclear Magnetic Resonance Spectroscopy: Bruker AV600 MHz nuclear magnetic resonance (NMR) spectrometer was used to record ^{1}H or ^{13}C NMR spectra at 80 °C or 60 °C using deuterated toluene (Tol-d_8) or deuterated hexafluoroisopropanol (HFIP-d_2) with tetramethylsilane (TMS) as the internal standard. The diffusion-ordered spectroscopy (DOSY) is

performed on a Bruker 600 MHz liquid NMR spectrometer at 298.1 K. 32 gradient strengths varying linearly between 5% and 100% of the maximum gradient strength and 16 scans per increment were used to construct the decay function. D20 = 1.2000 s, P30 = 1500.00 μs. The phase of the original data is adjusted manually and the baseline is smoothed via TopSpin 4.2.0. Bayesian method is used for processing the raw spectra. Resolution factor = 5.00 and repetitions = 3.

Solid-state Nuclear Magnetic Resonance Spectroscopy. One-dimensional ^{1}H magic angle spinning (MAS) and ^{13}C cross-polarization (CP) MAS solid-state NMR spectra were recorded on Bruker AVANCE III spectrometers operating at 600 MHz resonance frequencies for ^{1}H utilizing a 3.2 mm double-resonance probe. Dry nitrogen gas was utilized for sample spinning to prevent the degradation of the samples. NMR chemical shifts are reported with respect to the external references TMS and adamantane.

The 2D ^{1}H−^{13}C heteronuclear correlation (HETCOR) solid-state NMR spectroscopy experiments were conducted on a Bruker AVANCE III spectrometer using a 3.2 mm MAS probe. The experiments were performed according to the following scheme: 90° proton pulse, t_1 evolution period, CP to ^{13}C, and detection of the ^{13}C magnetization under TPPM decoupling. For the cross-polarization step, a ramped radio frequency (RF) field centered at 75 kHz was applied to the protons, while the ^{13}C channel RF field was matched to obtain an optimal signal. A total of 64 t_1 increments with 1024 scans each were collected. The sample spinning frequency was 8 kHz. Using a short contact time (500 μs) for the CP step, the polarization transfer in the dipolar correlation experiment was verified to be selective for the first coordination sphere to lead to correlations only between pairs of attached ^{1}H−^{13}C spins (C−H directly bonded).

For ^{13}C CP−MAS NMR experiments, the following sequence was used: 90° pulse on the proton (pulse length 2.5 μs), then a cross-polarization step with a contact time of typically 1 ms, and finally acquisition NMR signal under high-power proton decoupling. The delay between the scans was set to 4 s to allow the complete relaxation of the ^{1}H nuclei, and the number of scans ranged between 1024 and 4096 for ^{13}C and 32 for ^{1}H. An exponential apodization function corresponding to a line broadening of 80 Hz was applied prior to the Fourier transformation.

The 1D insensitive nuclei enhanced by polarization transfer (INEPT) spectra were recorded using a 2 s recycle delay, 20 ms acquisition time, and accumulation of 4k scans. ^{1}H and ^{13}C pulses were applied with a field strength of 70 and 50 kHz, respectively.

The solid-state $^{1}H-^{13}C$ WISE 2D NMR spectrum was performed on Bruker AVANCE III spectrometers operating at 600 MHz resonance frequencies using a 3.2 mm MAS probe. A MAS of 6 kHz was applied in all experiments. The increment time t_1 was set to 5 μs. the contact time was 1 ms.

SEC: For polyethylene precursor, high-temperature size exclusion chromatography measurements were carried out on the Agilent 1260 II infinity High-Temperature SEC (Agilent Technologies) with 2 × PLgel 10 μm MIXED-B, 300 × 7.5 mm columns. TCB was used as an eluent at a temperature of 150 °C. For $PE_{1.5k}$-based multiblock polymers, size exclusion chromatography (SEC) measurements equipped with a refractive index (RI) detector were conducted in THF at 50 °C using two identical PLgel MIXED-C columns (5 μm) at a flow rate of 1.0 mL min^{-1} (polystyrene standard).

DSC: Differential scanning calorimetry (DSC) measurements were performed at a heating/cooling rate of 10 °C min^{-1} on a Discovery 2500 DSC machine (TA instruments) under nitrogen with a flow rate of 50 mL min^{-1}. A heating/cooling rate of 5 °C min^{-1} may be needed for samples with a PLLA block.

TGA: Thermogravimetric analysis (TGA) was conducted on a Discovery 5500 TGA analyzer (TA Instruments) under nitrogen with a flow rate of 50 mL min^{-1} and a heating rate of 10 °C min^{-1}.

MALDI-TOF MS: MALDI-TOF MS analysis was performed on a Bruker Autoflex III Smartbeam MALDI-TOF mass spectrometer (Bruker, Germany). The samples were prepared according to ref.[1].

XRD: Wide-angle X-ray diffractions were carried out on Bruker D2 Phaser XRD spectrometry. The sample was pre-annealed at 180 °C (230 °C for samples with PGA block) for 5 minutes and slowly cooled down to 0 °C (~ 2 °C min^{-1}).

FTIR: Fourier-transform infrared spectroscopy (FTIR) spectrum was recorded on a Nicolet iS10 instrument (Thermo Scientific Corporation) machine ranging from 550 cm^{-1} to 4000 cm^{-1}.

Figures and Tables

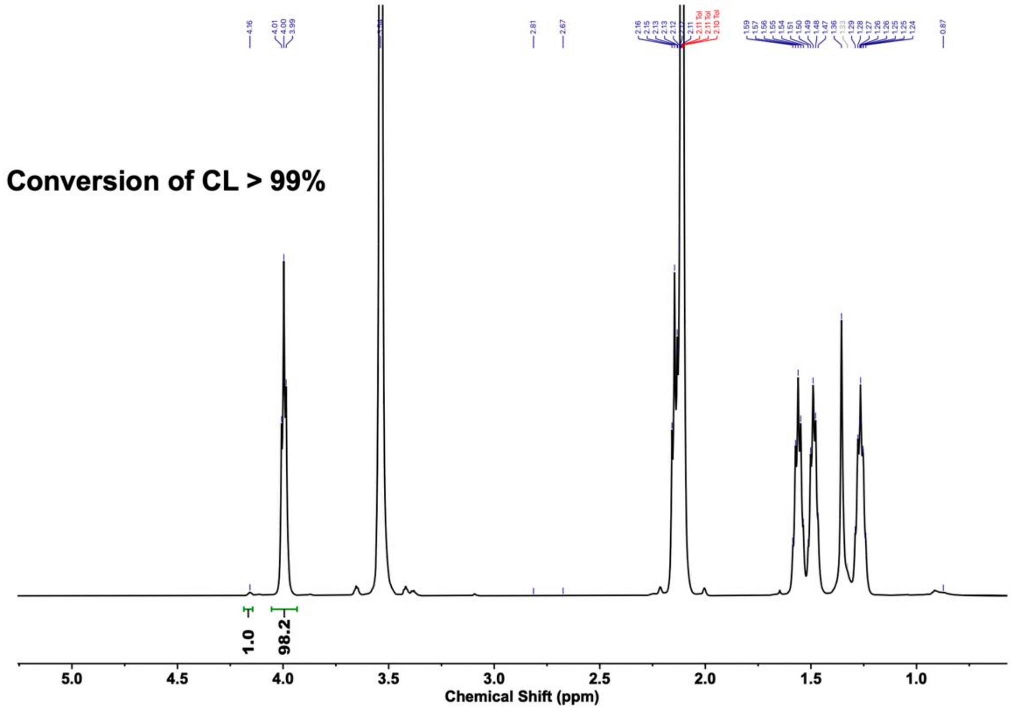

Supplementary Figure 1. ^{1}H NMR spectrum of PE-*b*-PEO-*b*-PCL-1 after polymerization for 45 hours (Table 1, entry 3, 600 MHz, 80 °C, toluene-d_8). The conversion of CL is higher than 99%.

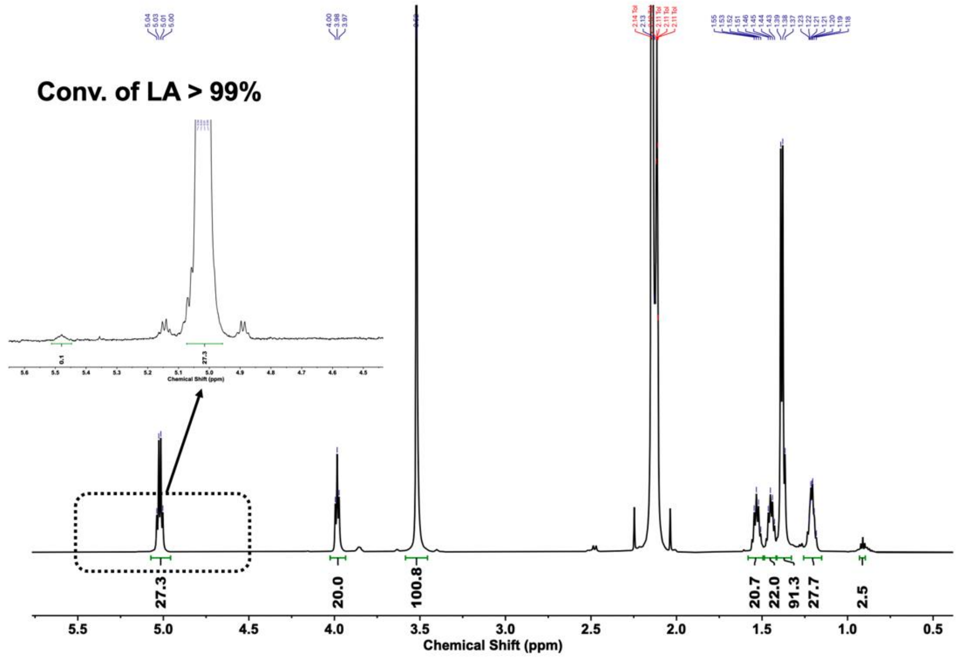

Supplementary Figure 2. ^{1}H NMR spectrum of PE-*b*-PEO-*b*-PCL-*b*-PLLA-1 after polymerization for 24 hours (Table 1, entry 4, 600 MHz, 80 °C, toluene-d_8). The conversion of LA is higher than 99%.

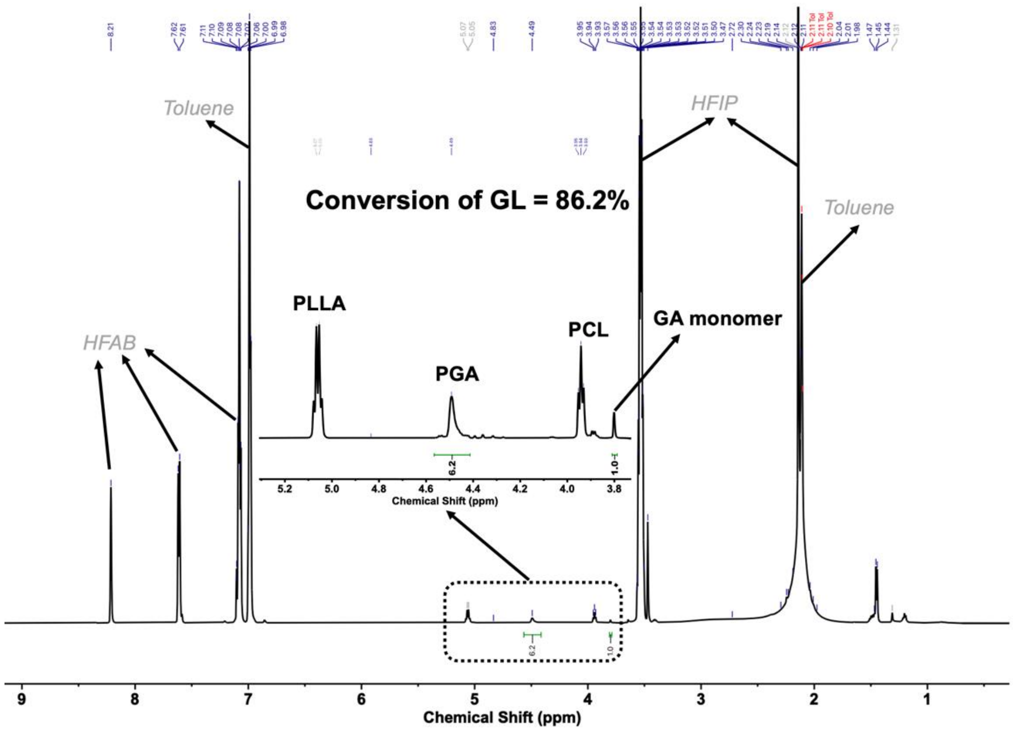

Supplementary Figure 3. ^{1}H NMR spectrum of PE-*b*-PEO-*b*-PCL-*b*-PLLA-*b*-PGA-1a with HFAB after polymerization for 20 hours (Table 1, entry 5, 600 MHz, 80 °C, toluene-d_8 and HFIP-d_2). The conversion of GA is 86.2%.

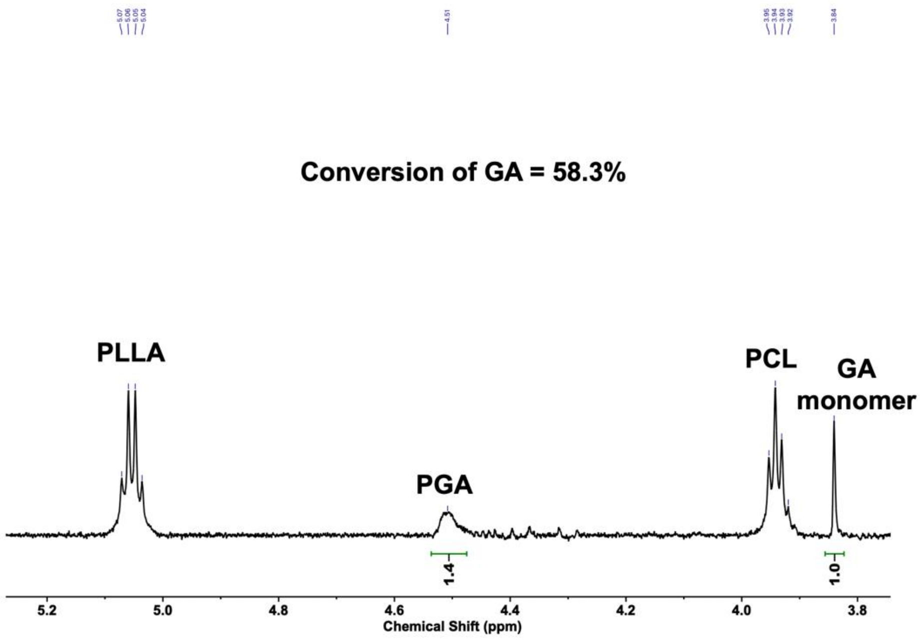

Supplementary Figure 4. ^{1}H NMR spectrum of PE-*b*-PEO-*b*-PCL-*b*-PLLA-*b*-PGA-1b without HFAB after polymerization for 36 hours (600 MHz, 80 °C, toluene-d_8 and HFIP-d_2). The conversion of GA is only 58.3%.

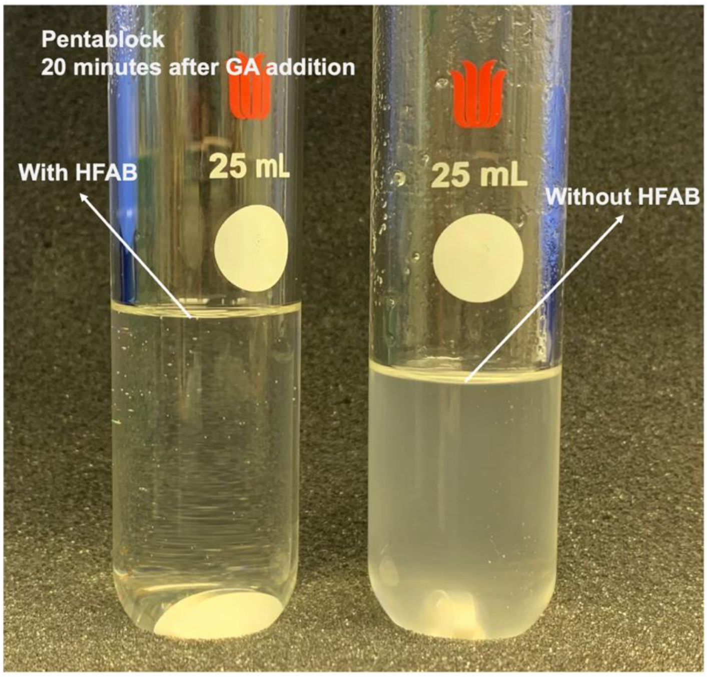

Supplementary Figure 5. A photo of the reaction mixture with (pentablock-1a) or without HFAB (pentablock-1b) 20 minutes after GA addition. The one without HFAB has already turned translucent.

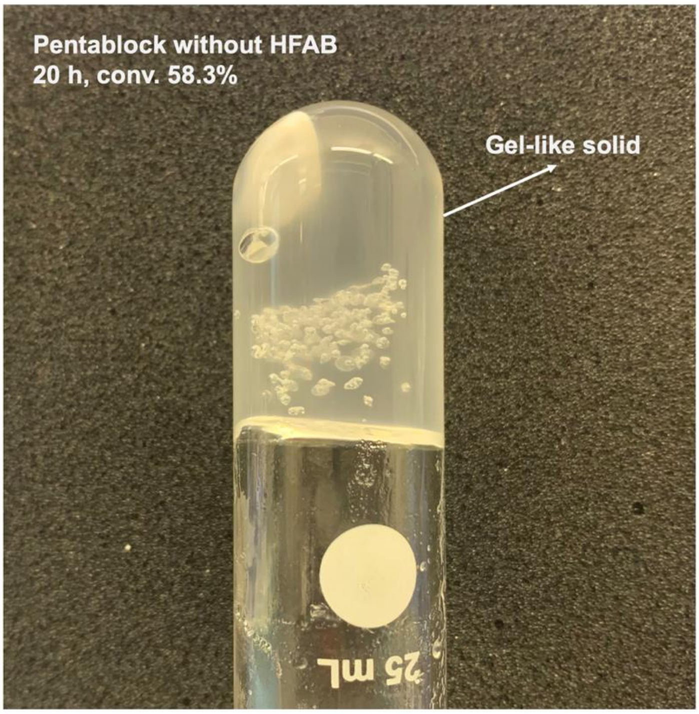

Supplementary Figure 6. A photo of the pentablock reaction mixture without HFAB (pentablock-1b) 20 hours after GA addition. It already became a gel-like solid.

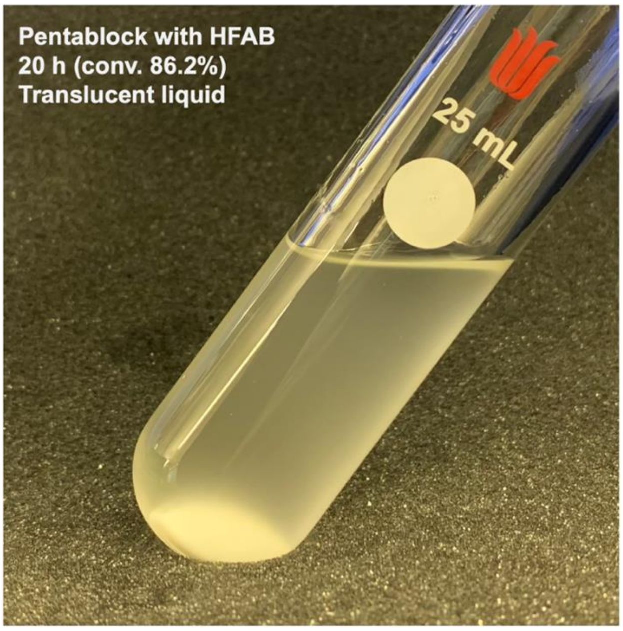

Supplementary Figure 7. A photo of the reaction mixture with HFAB (pentablock-1a) 20 hours after GA addition.

Supplementary Table 1. Molecular weight and thermal properties of the pentablock quintopolymer (pentablock-2) and corresponding precursors.[a]

Entry	Sample	t[c] (h)	Conv. (%)[d]	$M_{n,NMR}$[e] (kg mol^{-1})		T_m/T_c[g] (ºC)				
				each block	total[f]	PE	PEO	PCL	PLLA	PGA
1	PE-OH$_{7k}$[b]	18	>99	7.0	7.0	131.8/ 118.7				
2	PE-*b*-PEO-2	15	>99	10.6	17.6	131.2/ 118.6	61.9/ 41.8			
3	PE-*b*-PEO-*b*-PCL-2	45	>99	11.4	29.0	131.1/ 117.8	56.2/ 29.6	56.2/ 44.0		
4	PE-*b*-PEO-*b*-PCL-*b*-PLLA-2	24	>99	25.9	54.9	128.8/ 96.6	39.9/ 36.4	39.9/ 36.4	155.7/ 114.3	
5	PE-*b*-PEO-*b*-PCL-*b*-PLLA-*b*-PGA-2	20	-[h]	-[h]	-[h]	126.7/ 90.5	41.8/ 42.0	41.8/ 42.0	147.6/ 119.1	209.0/ 144.3

[a] Polymerization was performed with the PE-OH macroinitiator at 80 ºC in toluene via ^{t}BuP$_4$/DPP/Sn(Oct)$_2$ catalyst switch strategy. The targeting molecular weight for each block is 7 kg mol^{-1}, 10 kg mol^{-1}, 10 kg mol^{-1}, 25 kg mol^{-1}, and 15 kg mol^{-1}, respectively. [b] Prepared by polyhomologation of sulfoxonium methylene in toluene at 80 ºC. [c] Reaction time for the last block. [d] Conversion of the last block, determined by ^{1}H NMR at 60 ºC (600 MHz, toluene-d_8), except for the PGA block, where a mixture of toluene-d_8/HFIP-d_2 (~7/3 v/v) was used. [e] Determined by ^{1}H NMR of the purified sample at 60 ºC (600 MHz, toluene-d_8), except for the PE-OH$_{7k}$ precursor, where a higher temperature of 90 ºC was used. [f] Total $M_{n,NMR}$ at each stage. [g] Determined by DSC (10 ºC min^{-1} for heating and cooling, under N_2 atmosphere). [h] The sample is not fully soluble, even in the mixture of HFAB and toluene at high temperatures.

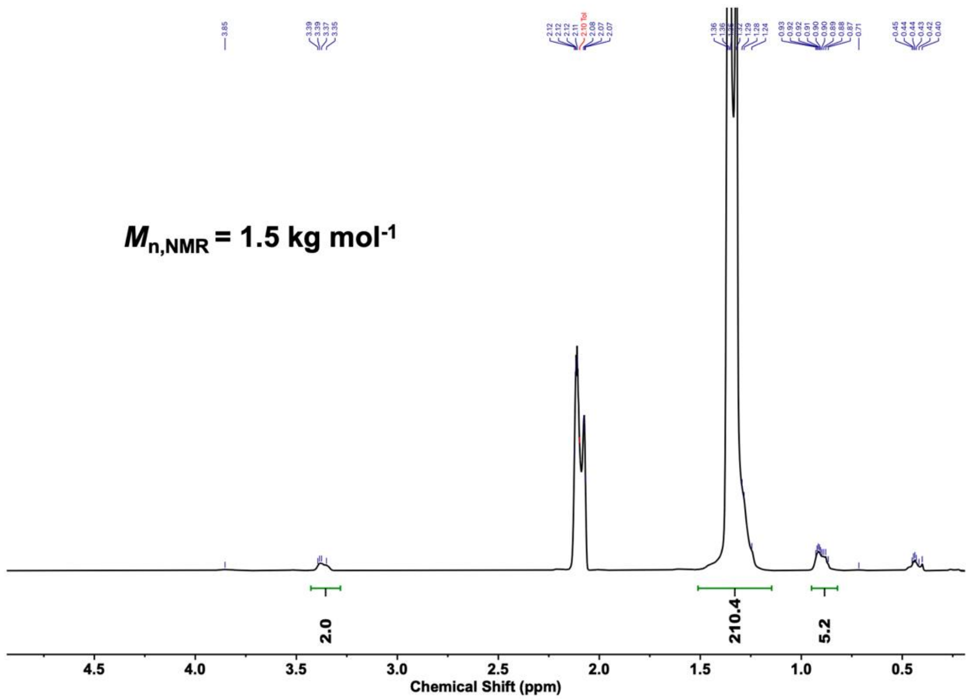

Supplementary Figure 8. ^{1}H NMR spectrum of isolated PE-$OH_{1.5k}$ (Table 1, entry 1, 600 MHz, 80 °C, toluene-d_8). The $M_{n,NMR}$ of this sample is 1.5 kg mol^{-1}.

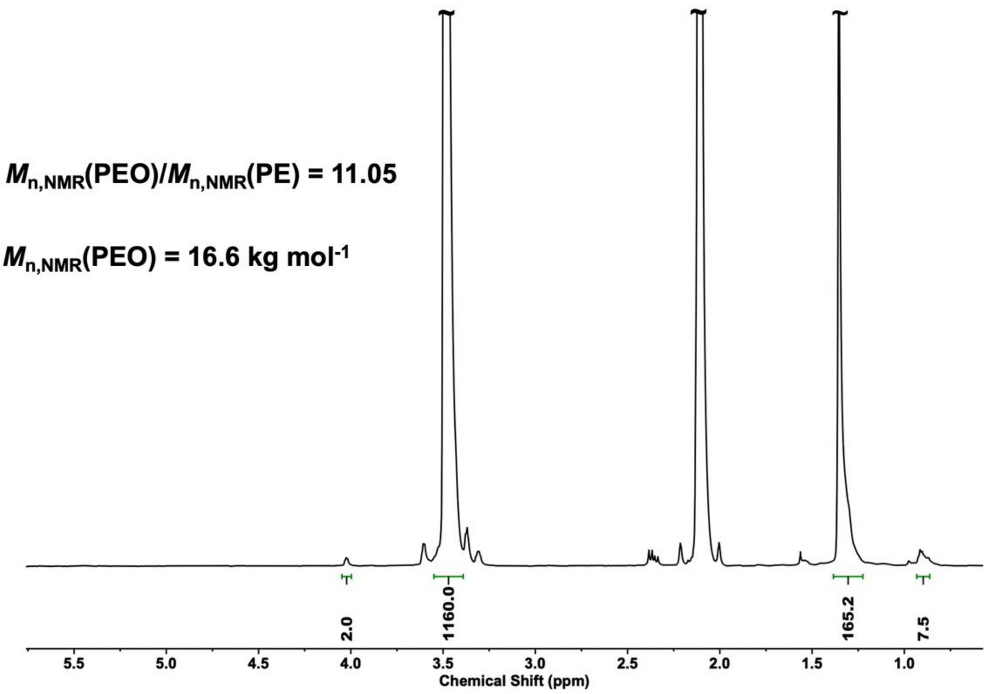

Supplementary Figure 9. ^{1}H NMR spectrum of isolated PE-*b*-PEO-1 (Table 1, entry 2, 600 MHz, 60 °C, toluene-d_8). The $M_{n,NMR}$ of the PEO block is 16.6 kg mol^{-1}.

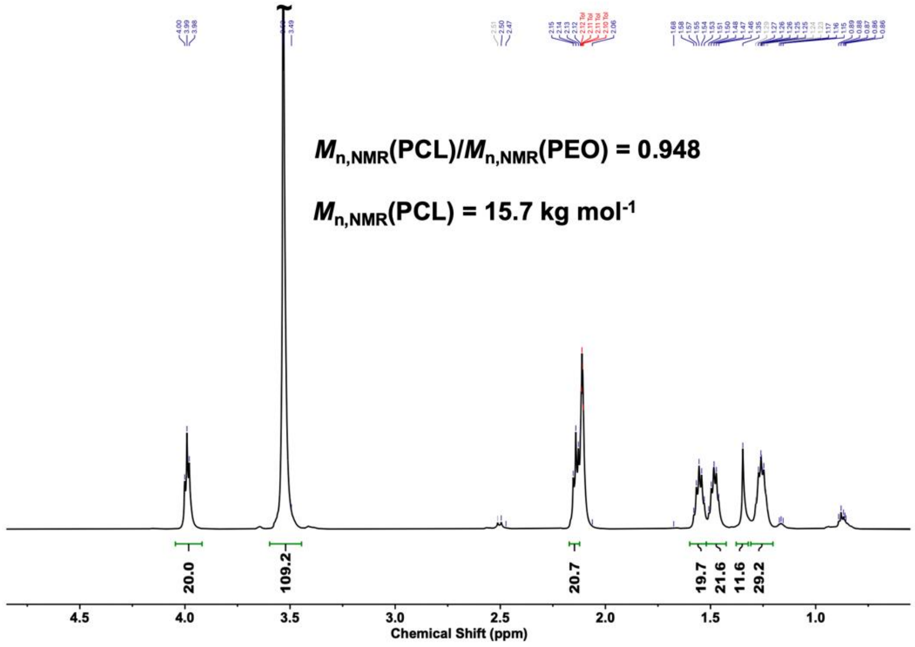

Supplementary Figure 10. ^{1}H NMR spectrum of isolated PE-*b*-PEO-*b*-PCL-1 (Table 1, entry 3, 600 MHz, 60 °C, toluene-d_8). The $M_{n,NMR}$ of the PCL block is 15.7 kg mol^{-1}.

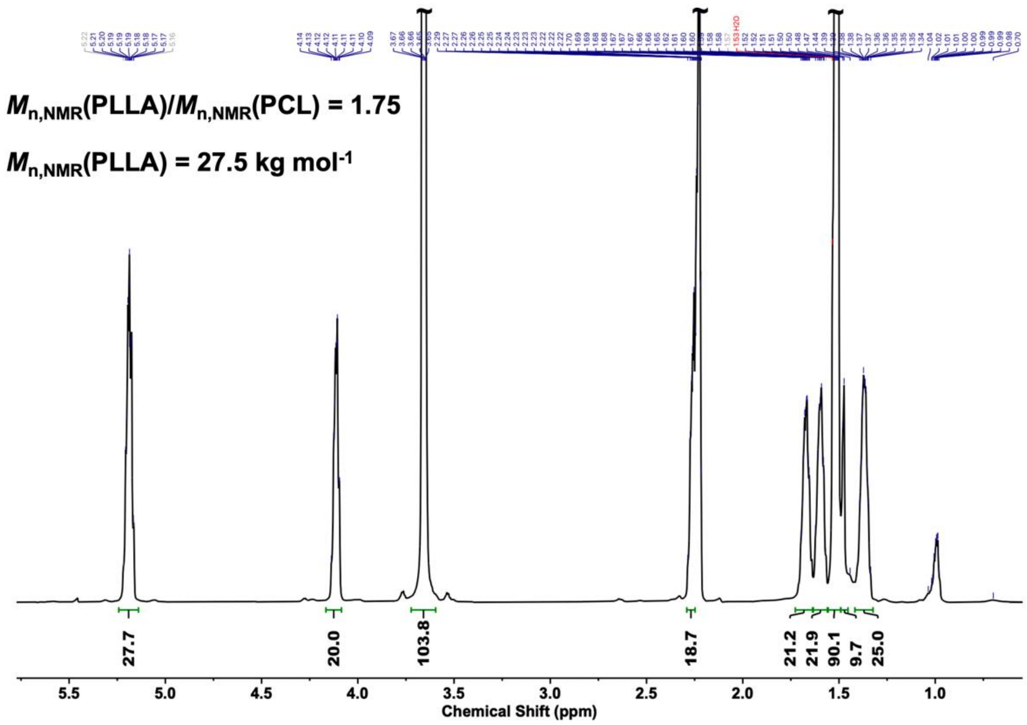

Supplementary Figure 11. ^{1}H NMR spectrum of isolated PE-*b*-PEO-*b*-PCL-*b*-PLLA-1 (Table 1, entry 4, 600 MHz, 60 °C, toluene-d_8). The $M_{n,NMR}$ of the PLLA block is 27.5 kg mol^{-1}.

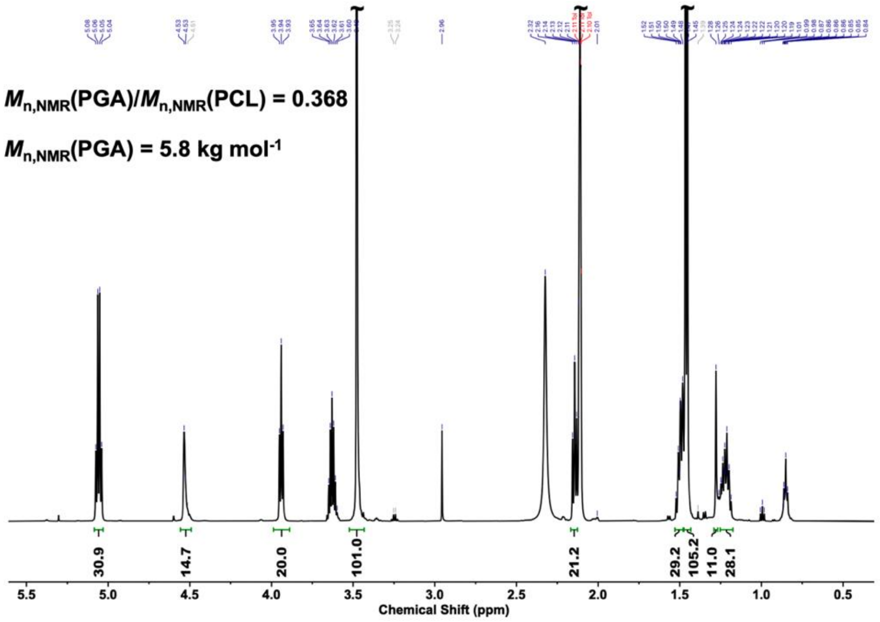

Supplementary Figure 12. ^{1}H NMR spectrum of isolated PE-*b*-PEO-*b*-PCL-*b*-PLLA-*b*-PGA-1a (Table 1, entry 5, 600 MHz, 60 °C, toluene-d_8, and HFIP-d_2). The $M_{n,NMR}$ of the PGA block is 5.8 kg mol^{-1}.

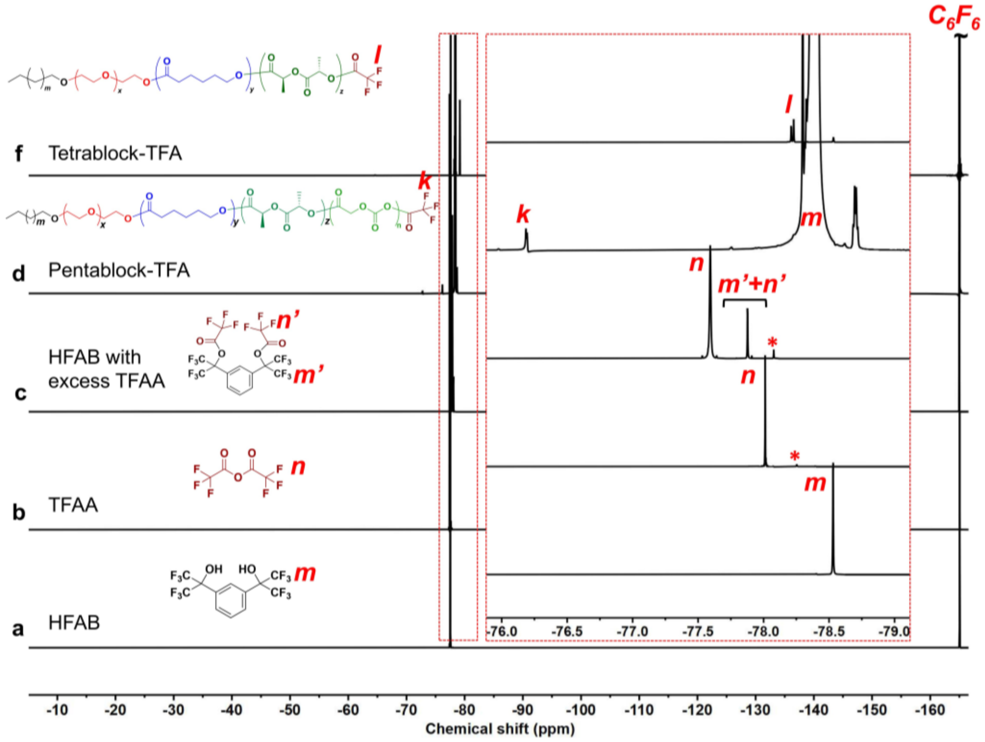

Supplementary Figure 13 ^{19}F NMR spectra of **a** HFAB, **b** TFAA, **c** HFAB reacted with a large excess of TFAA, **d** pentablock-TFA in the presence of HFAB, and **e** tetrablock-TFA (800 MHz, $CDCl_3$, room temperature (RT), hexaflurobenzene (C_6F_6) was used as internal standard). * signal corresponds to trifluoroacetic acid.

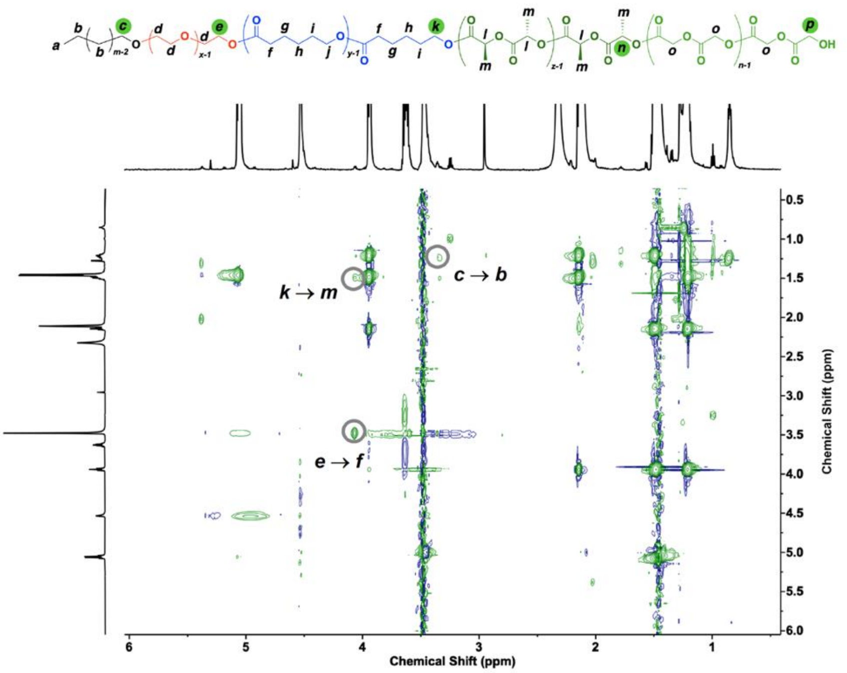

Supplementary Figure 14. ^{1}H–^{1}H TOCSY spectrum of isolated PE-*b*-PEO-*b*-PCL-*b*-PLLA-*b*-PGA-1a (Table 1, entry 5, 600 MHz, 60 °C, toluene-d_8 and HFIP-d_2).

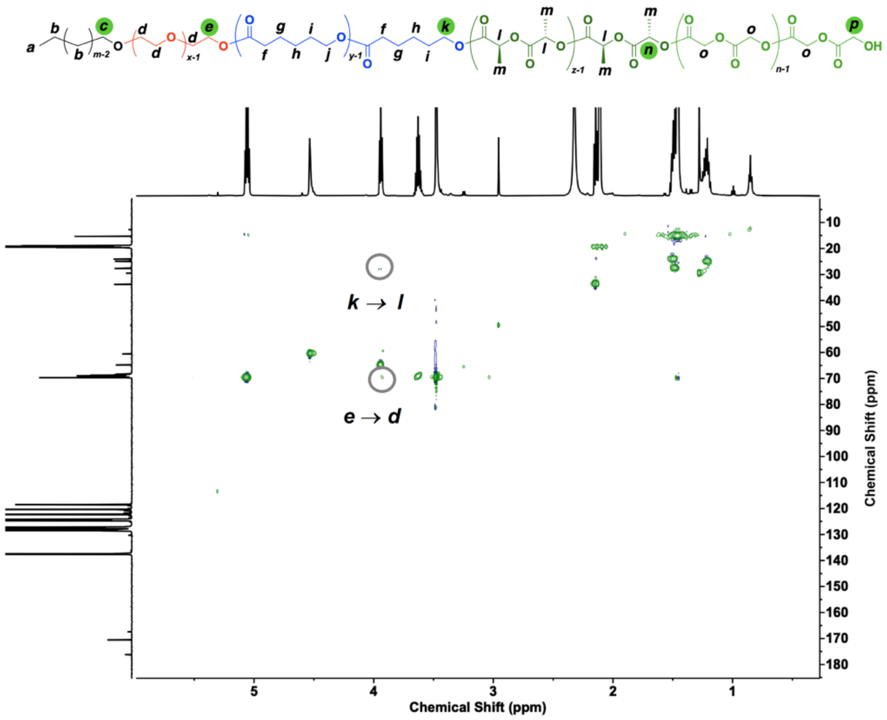

Supplementary Figure 15. ^{1}H–^{13}C HSQC spectrum of isolated PE-*b*-PEO-*b*-PCL-*b*-PLLA-*b*-PGA-1a (Table 1, entry 5, 600 MHz, 60 °C, toluene-d_8 and HFIP-d_2).

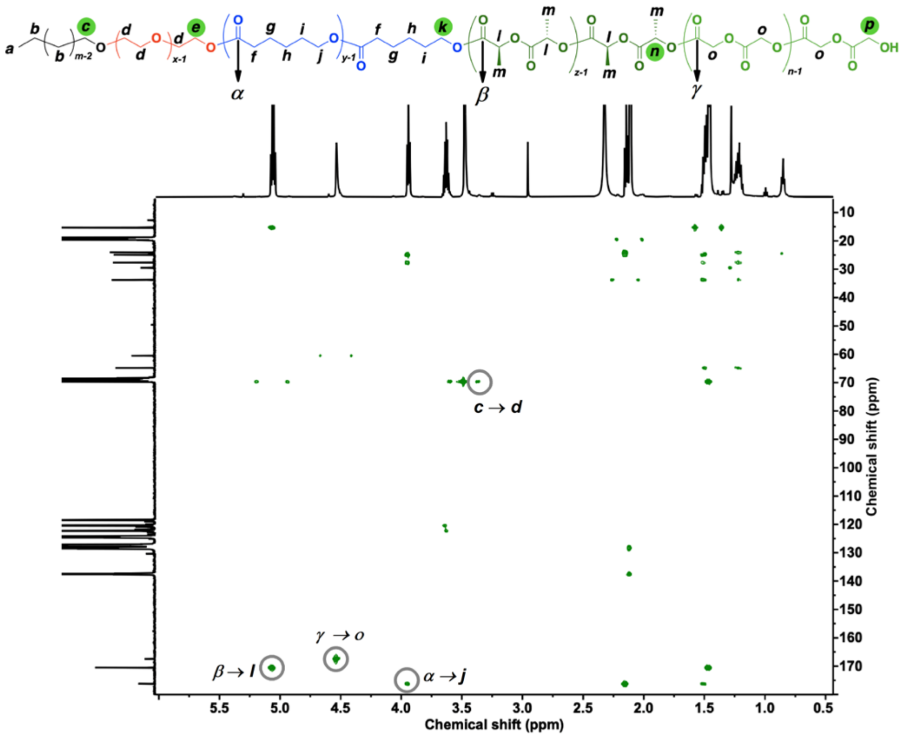

Supplementary Figure 16. ^1H–^{13}C HMBC spectrum of isolated PE-*b*-PEO-*b*-PCL-*b*-PLLA-*b*-PGA-1a (Table 1, entry 5, 600 MHz, 60 °C, toluene-d_8 and HFIP-d_2).

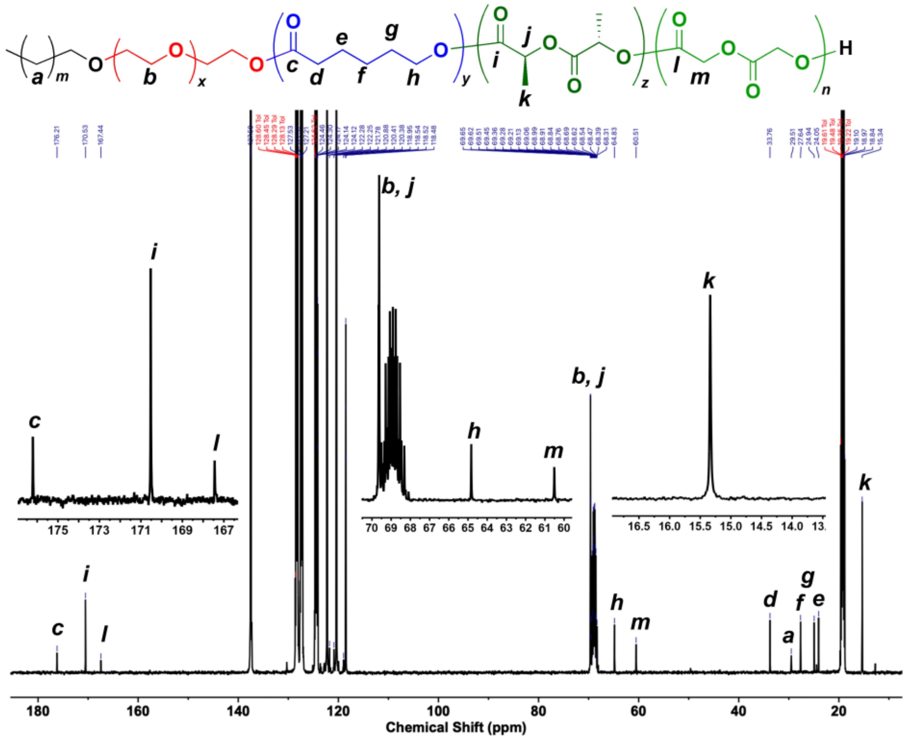

Supplementary Figure 17. ^{13}C NMR spectrum of isolated PE-*b*-PEO-*b*-PCL-*b*-PLLA-*b*-PGA-1a (Table 1, entry 5, 150 MHz, 60 °C, toluene-d_8 and HFIP-d_2).

Supplementary Discussion: Solid-state NMR analysis

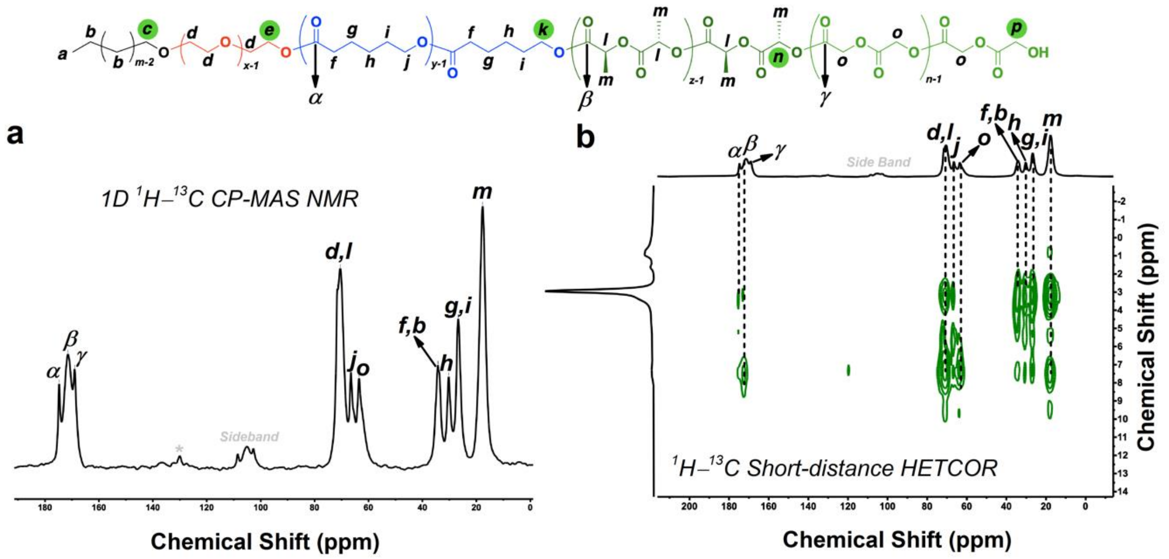

Supplementary Figure 18. a 1D ^1H–^{13}C CP MAS NMR spectrum and **b** ^1H–^{13}C short distance HETCOR solid-state NMR spectrum of PE-*b*-PEO-*b*-PCL-*b*-PLLA-*b*-PGA-1a (600 MHz, RT). The chemical shift was referenced to adamantane.

The structure of the PE-*b*-PEO-*b*-PCL-*b*-PLLA-*b*-PGA was also confirmed by solid-state NMR. ^1H magic angle spinning (MAS) NMR spectrum and 1D ^1H–^{13}C cross-polarization (CP) MAS NMR spectrum (**Supplementary Figure 18**-**20** for pentablock-1a; **Supplementary Figure 21-22** for pentablock-2) were first analyzed. The -CH_2- signal of the PE block was found at 0.73 ppm on the ^1H spectrum and 33.8 ppm on the ^{13}C spectrum. The correlation of these two signals was confirmed by the 2D ^1H–^{13}C short-distance heteronuclear correlation spectroscopy (HETCOR, **Supplementary Figure 18b**). For the PEO block, the correlation of the proton signal at 3.0 ppm and the carbon signal at 71.5 ppm was also found on the HETCOR. The correlation signal was shown for the PCL block at 3.42 ppm and 66.5 ppm, which corresponds to the -C=O(CH_2)CH_2O- and -C=O(CH_2)CH_2O-. The other signal of PCL was found on HETCOR as well, which is consistent with the HMBC spectrum. Similarly, the carbonyl signal of PLLA and PGA was also found to correlate with the corresponding proton signals at 5.4 ppm and 4.5 ppm, respectively.

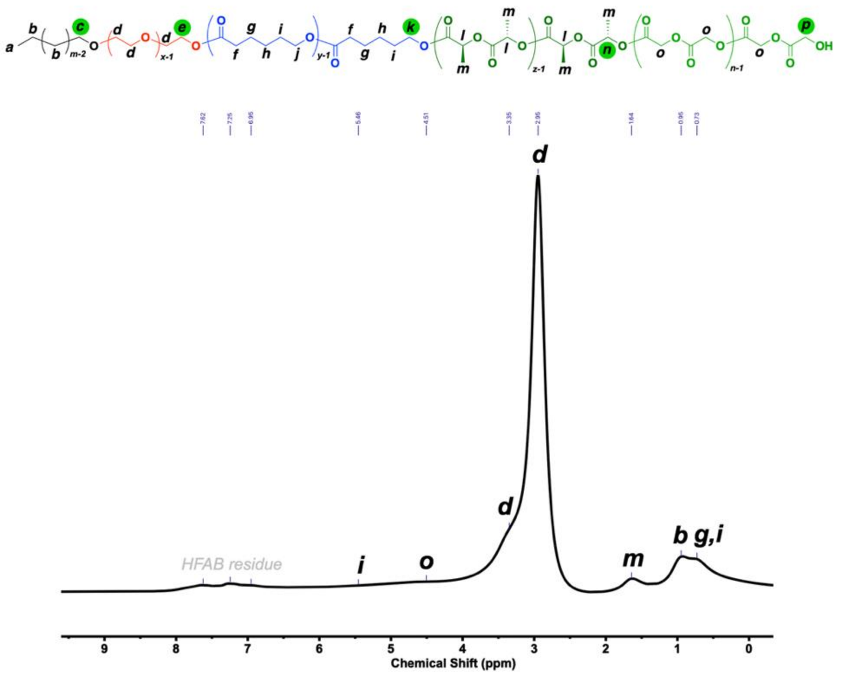

Supplementary Figure 19. Solid-state ^1H MAS NMR spectrum of the PE-*b*-PEO-*b*-PCL-*b*-PLLA-*b*-PGA-1a (pentablock-1a, Table 1, entry 5). (600 MHz, RT)

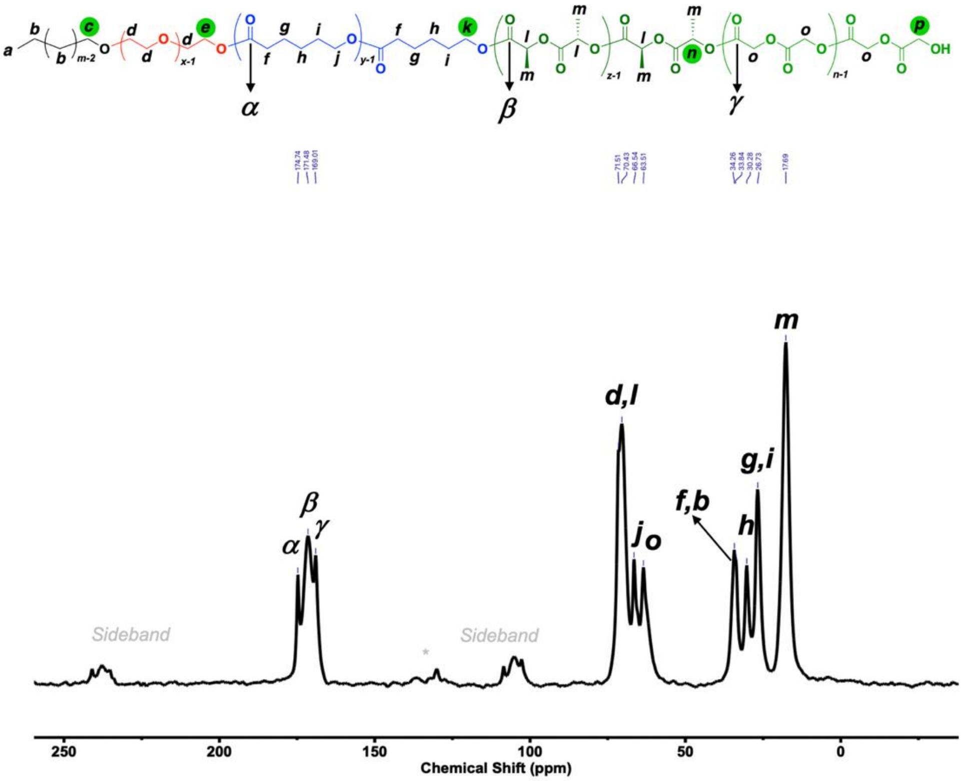

Supplementary Figure 20. Solid-state ^1H–^{13}C CP-MAS NMR spectrum of the PE-*b*-PEO-*b*-PCL-*b*-PLLA-*b*-PGA-1a (pentablock-1a, Table 1, entry 5). (150 MHz, RT)

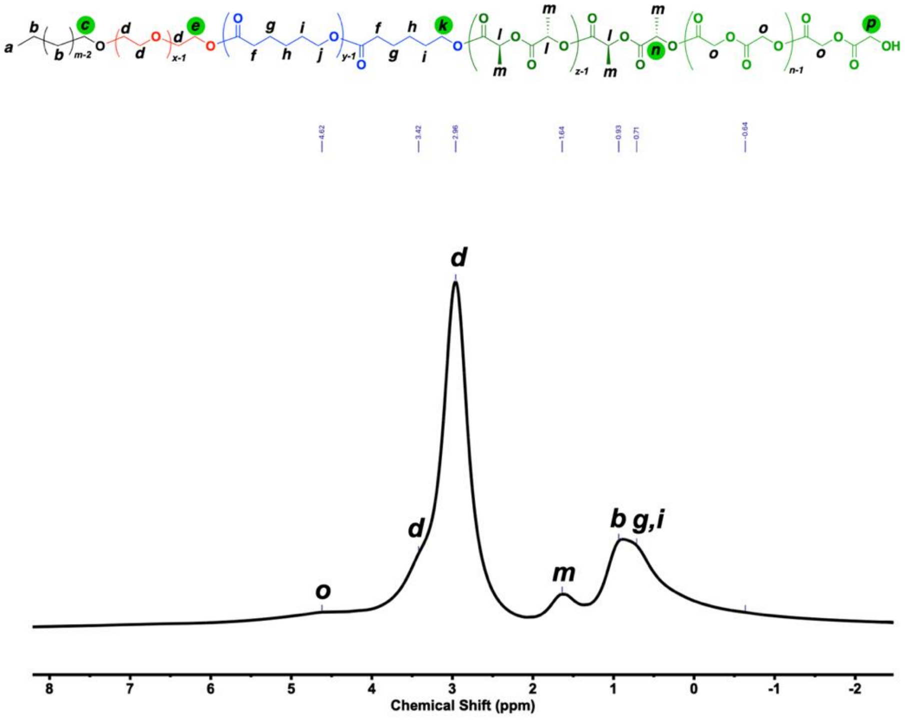

Supplementary Figure 21. Solid-state ^{1}H MAS NMR spectrum of the PE-*b*-PEO-*b*-PCL-*b*-PLLA-*b*-PGA-2 (pentablock-2, Table S1, entry 5). (600 MHz, RT)

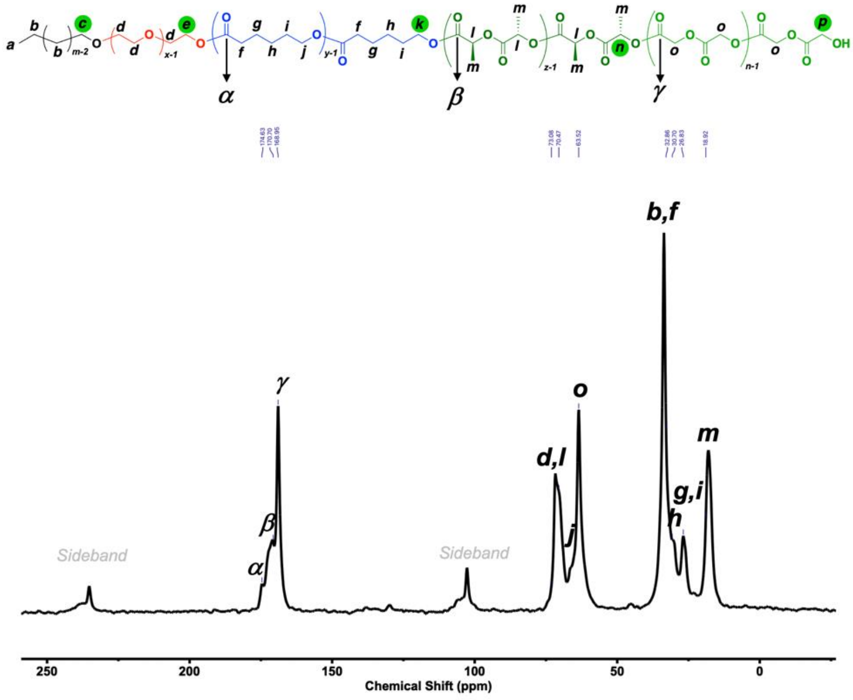

Supplementary Figure 22. Solid-state ^{1}H–^{13}C CP-MAS NMR spectrum of the PE-*b*-PEO-*b*-PCL-*b*-PLLA-*b*-PGA-2 (pentablock-2, Table S1, entry 5). (150 MHz, RT)

Supplementary Discussion: FITR analysis

The $-CH_2-$ stretching bands (2915.4 cm^{-1} and 2847.9 cm^{-1}) were clearly shown on the spectra of $PE\text{-}OH_{1.5k}$ (**e**, black, **Supplementary Figure 23**). The ether stretching band of PEO (-C-O-C-, 1095.4 cm^{-1}) was also shown on the spectrum of PE-*b*-PEO-1 (**d**, red). The characteristic C=O stretch band of PCL and PLLA were found at 1722.6 cm^{-1} (**c**, blue) and 1755.4 cm^{-1} (**b**, dark green), respectively. The overlapping of the carbonyl stretching band of PGA and PLLA was solved by subtracting the tetrablock spectrum (**b**, dark green) from the pentablock spectrum (**a**, light green), which resulted in the appearance of a new peak at 1743.9 cm^{-1} corresponding to PGA (**Supplementary Figure 25**).

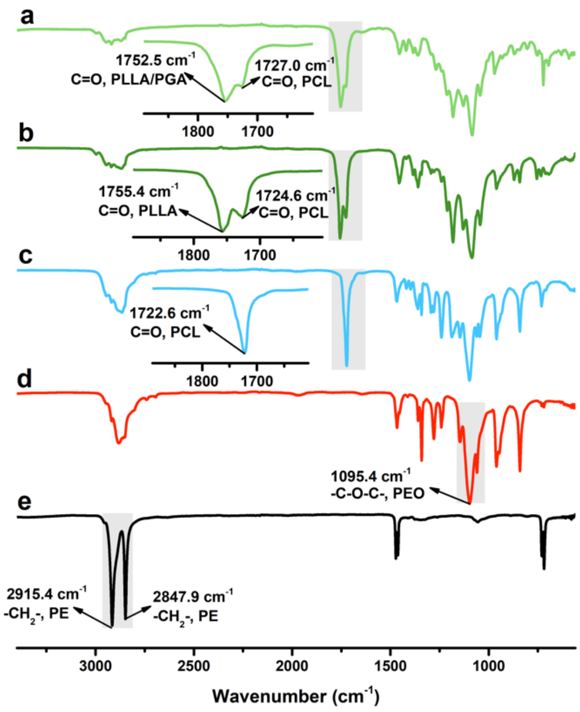

Supplementary Figure 23. FTIR spectra of the PE-*b*-PEO-*b*-PCL-*b*-PLLA-*b*-PGA-1a (pentablock-1, Table 1, entry 5, **a**, light green), PE-*b*-PEO-*b*-PCL-*b*-PLLA-1 (**b**, dark green), PE-*b*-PEO-*b*-PCL-1 (**c**, blue), PE-*b*-PEO-1 (**d**, red) and the PE-OH precursors ($PE\text{-}OH_{1.5k}$, **e**, black).

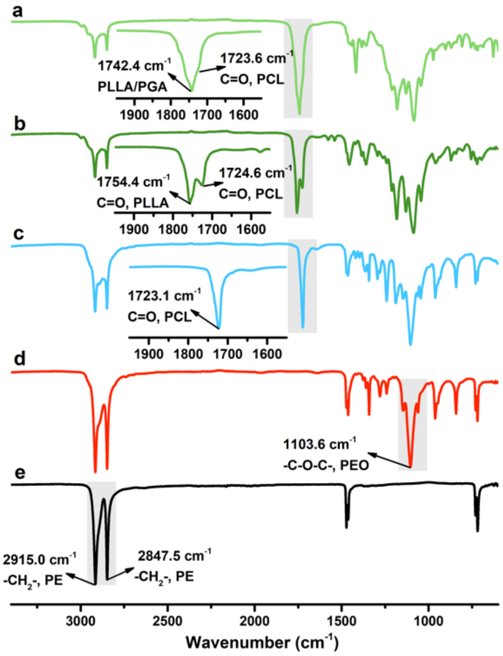

Supplementary Figure 24. FTIR spectra of the PE-*b*-PEO-*b*-PCL-*b*-PLLA-*b*-PGA-2 (pentablock-2, Table S1, entry 5, **a**, light green), PE-*b*-PEO-*b*-PCL-*b*-PLLA-2 (**b**, dark green), PE-*b*-PEO-*b*-PCL-2 (**c**, blue), PE-*b*-PEO-2 (**d**, red) and the PE-OH precursors (PE-OH_{7k}, **e**, black).

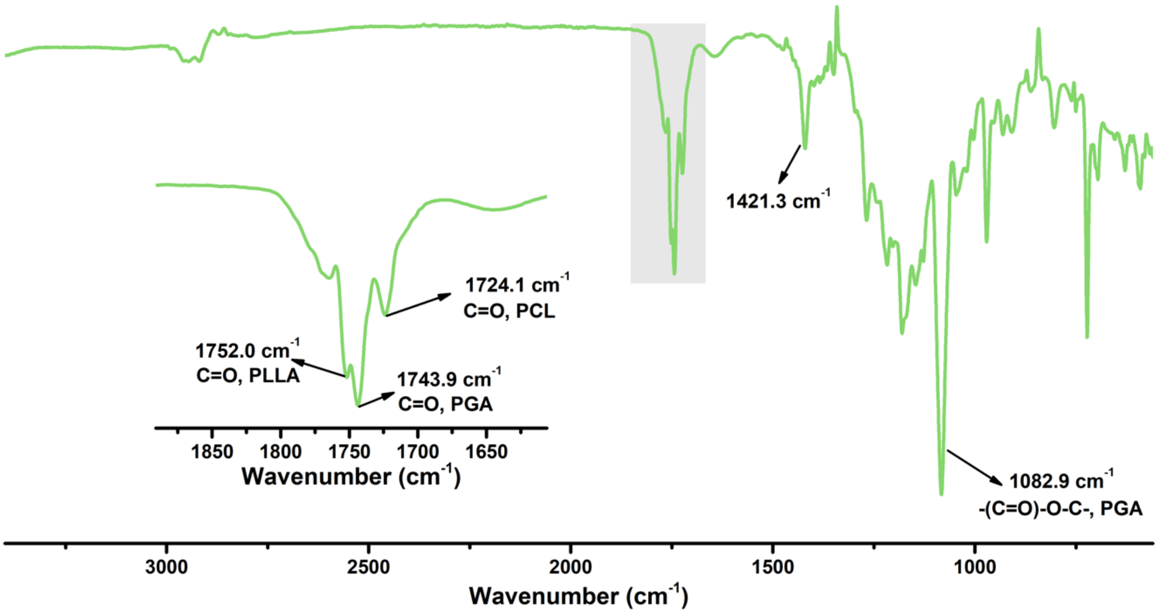

Supplementary Figure 25. FTIR spectrum of the PE-*b*-PEO-*b*-PCL-*b*-PLLA-*b*-PGA-1a (pentablock-1, Table 1, entry 5), after subtracting the PE-*b*-PEO-*b*-PCL-*b*-PLLA-1 spectrum.

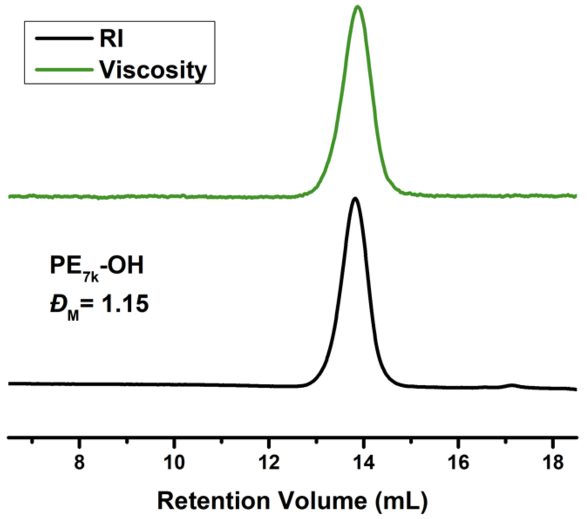

Supplementary Figure 26. HT-SEC traces of PE-OH_{7k} (Table 1, entry 1; TCB, 150 °C, PS standards)

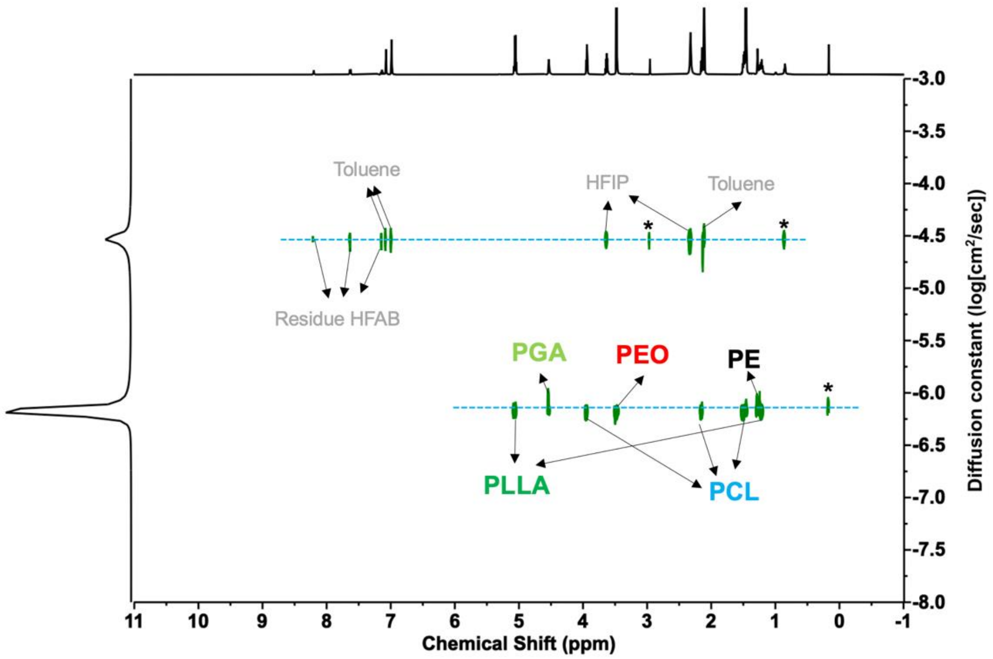

Supplementary Figure 27. DOSY spectrum of the PE-*b*-PEO-*b*-PCL-*b*-PLLA-*b*-PGA-1a (Table 1, entry 5, 600 MHz, 60 °C, toluene-d_8 and HFIP-d_2).

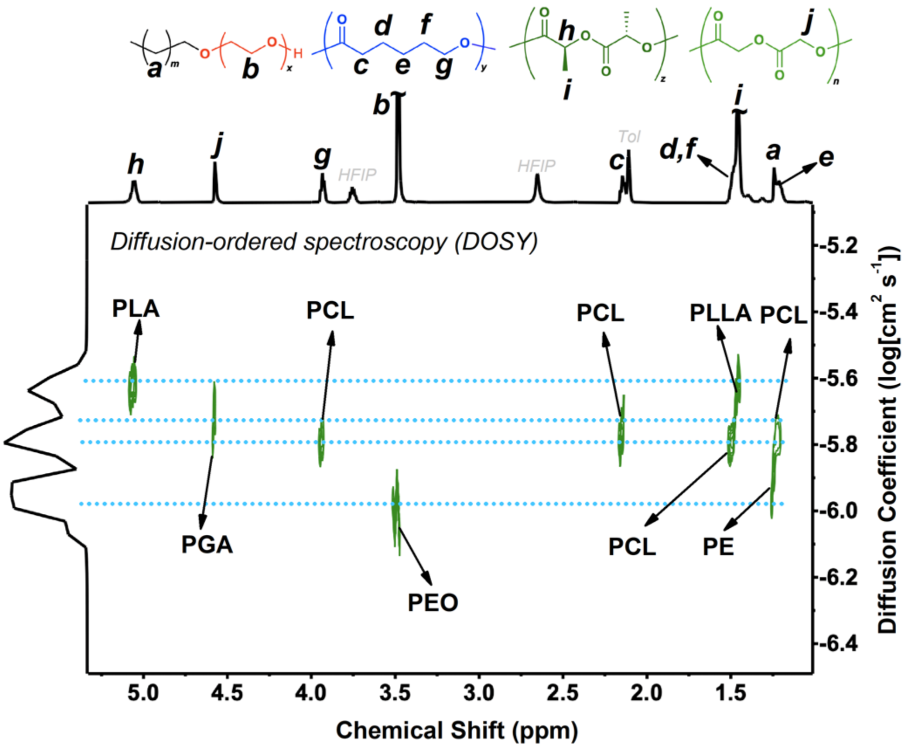

Supplementary Figure 28. DOSY spectrum of the mixture (physical blend) of PE-*b*-PEO-1, PCL, PLLA, and PGA (600 MHz, 60 °C, toluene-d_8 and HFIP-d_2).

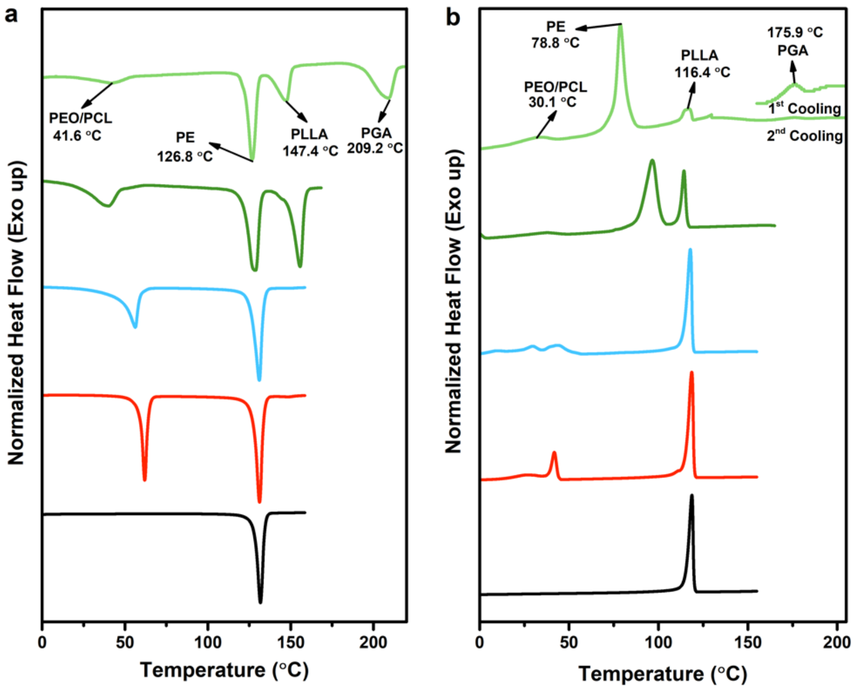

Supplementary Figure 29. DSC scans of PE-*b*-PEO-*b*-PCL-*b*-PLLA-*b*-PGA-2 (Supplementary Table 1, entry 5, light green) and all the precursors. a) Heating scan (10 °C min^{-1}). b) Cooling scan (10 °C min^{-1}).

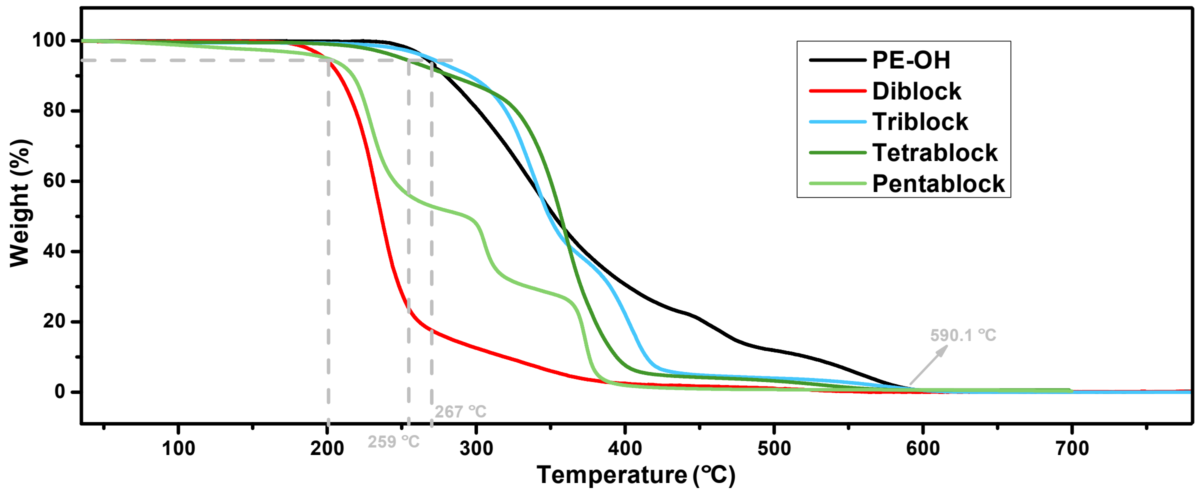

Supplementary Figure 30. TGA thermograms of the PE-*b*-PEO-*b*-PCL-*b*-PLLA-*b*-PGA-1a (pentablock-1a, Table 1, entry 5) and all corresponding precursors (heating rate: 10 °C min^{-1}).

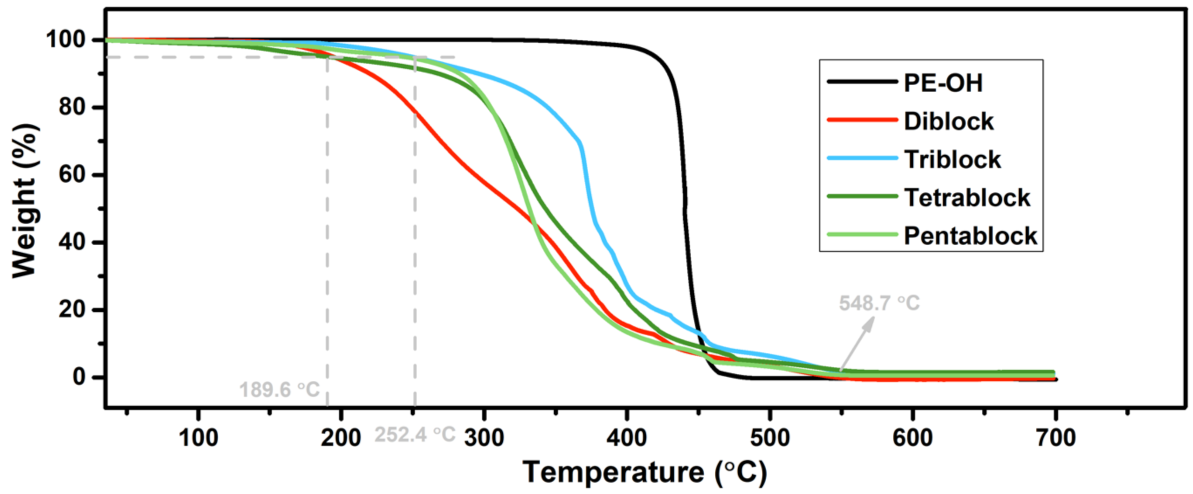

Supplementary Figure 31. TGA thermograms of the PE-*b*-PEO-*b*-PCL-*b*-PLLA-*b*-PGA-2 (pentablock-2, Table S1, entry 5) and all corresponding precursors (heating rate: 10 °C min^{-1}).

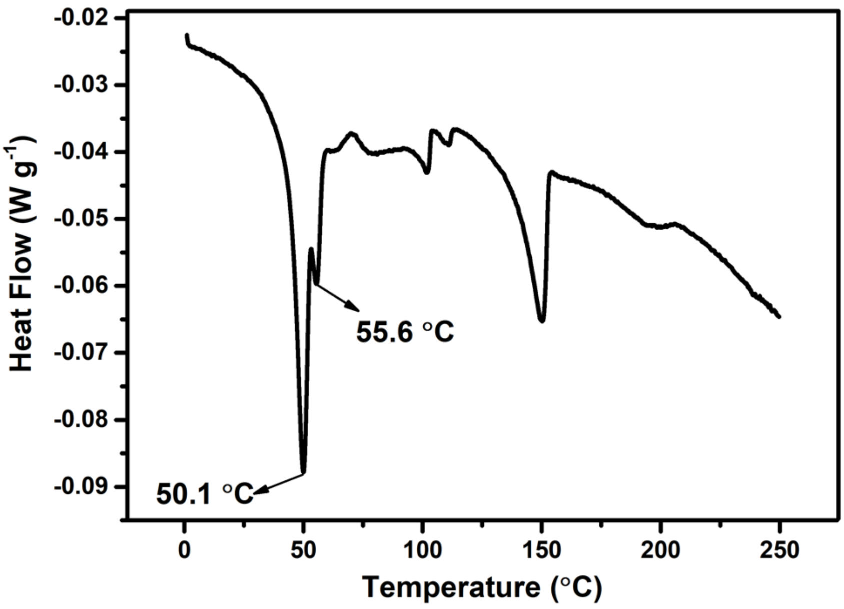

Supplementary Figure 32. DSC curve of PE-*b*-PEO-*b*-PCL-*b*-PLLA-*b*-PGA-1a. The heating rate was 1 °C min^{-1}.

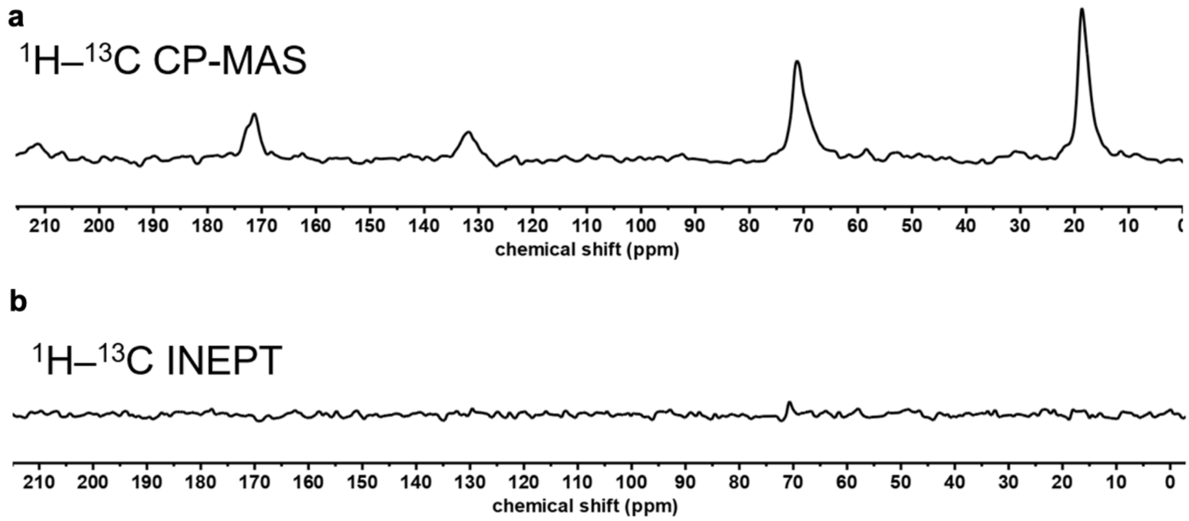

Supplementary Figure 33. **a** ^{1}H–^{13}C CP–MAS NMR spectrum and **b** INEPT NMR spectrum of a 1.4 kg mol^{-1} PLLA homopolymer at 120 °C.

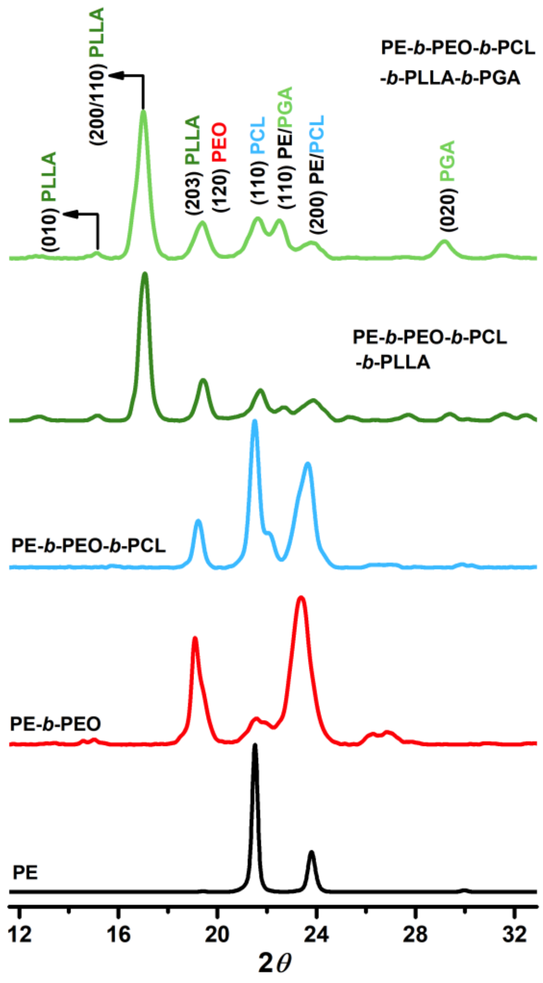

Supplementary Figure 34 | X-ray diffraction patterns of PE-b-PEO-b-PCL-b-PLLA-b-PGA-1a and its precursors. Pentabock-1a (light green), PE-*b*-PEO-*b*-PCL-*b*-PLLA-1 (dark green), PE-*b*-PEO-*b*-PCL-1 (blue), PE-*b*-PEO-1 (red) and the PE-$OH_{1.5k}$ precursors (black).

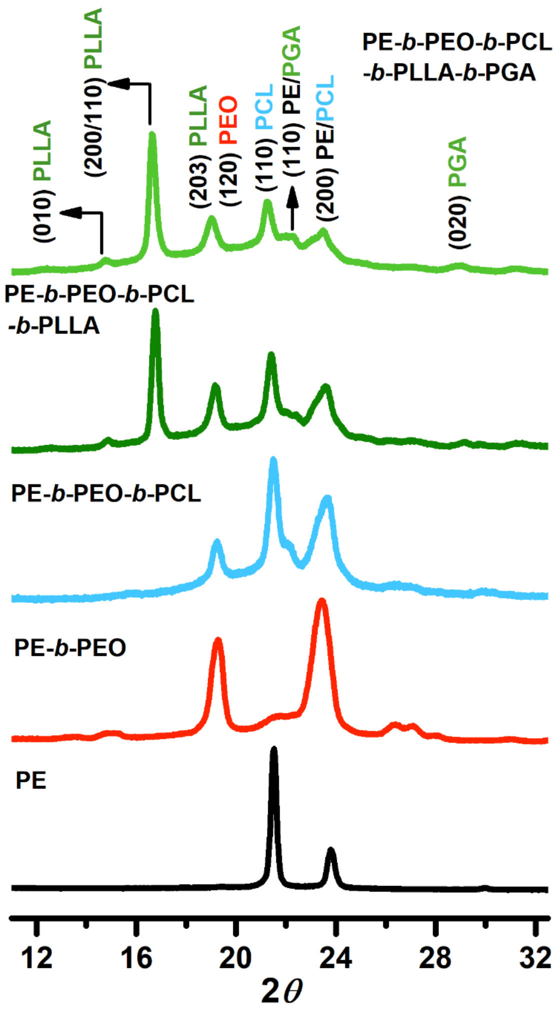

Supplementary Figure 35. X-ray diffraction patterns of PE-*b*-PEO-*b*-PCL-*b*-PLLA-*b*-PGA-1b (pentablock-1b, light green), PE-*b*-PEO-*b*-PCL-*b*-PLLA-1 (dark green), PE-*b*-PEO-*b*-PCL-1 (blue), PE-*b*-PEO-1 (red) and the PE-OH precursors (PE-$OH_{1.5k}$, black).

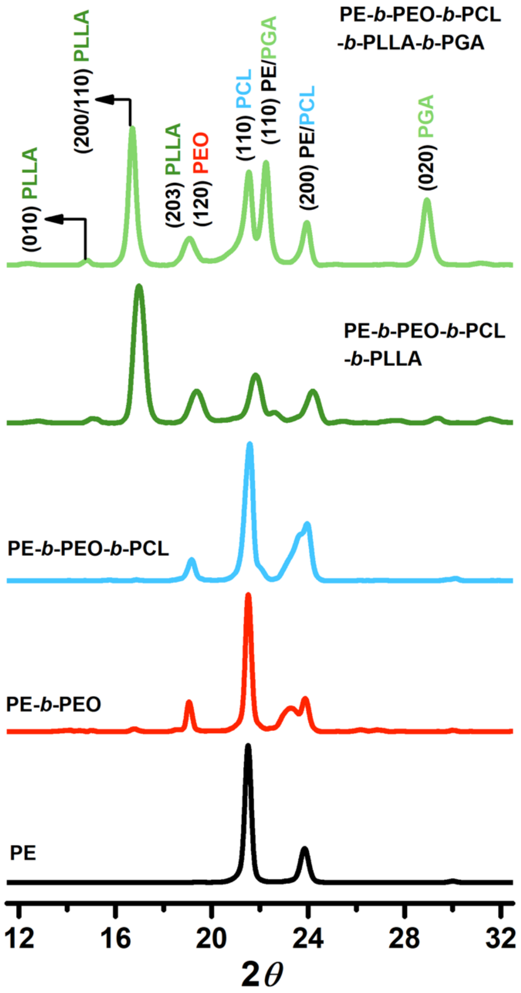

Supplementary Figure 36. X-ray diffraction patterns of PE-*b*-PEO-*b*-PCL-*b*-PLLA-*b*-PGA-2 (pentablock-2, Table S1, entry 5, light green), PE-*b*-PEO-*b*-PCL-*b*-PLLA-2 (dark green), PE-*b*-PEO-*b*-PCL-2 (blue), PE-*b*-PEO-2 (red) and the PE-OH precursors (PE-OH_{7k}, black).

References

1. Zhao J, Pahovnik D, Gnanou Y, Hadjichristidis N. Phosphazene-Promoted Metal-Free Ring-Opening Polymerization of Ethylene Oxide Initiated by Carboxylic Acid. *Macromolecules* 2014, **47**(5)**:** 1693-1698.
